# Identification of genes for small non-coding RNAs that belong to the regulon of the two-component regulatory system CiaRH in *Streptococcus*

**DOI:** 10.1186/1471-2164-11-661

**Published:** 2010-11-24

**Authors:** Patrick Marx, Michael Nuhn, Martá Kovács, Regine Hakenbeck, Reinhold Brückner

**Affiliations:** 1 Department of Microbiology, University of Kaiserslautern, D-67663 Kaiserslautern, Germany; 2 European Bioinformatics Institute, Wellcome Trust Genome Campus, Hinxton, Cambridge CB10 1SD, UK

## Abstract

**Background:**

Post-transcriptional regulation by small RNAs (sRNAs) in bacteria is now recognized as a wide-spread regulatory mechanism modulating a variety of physiological responses including virulence. In *Streptococcus pneumoniae*, an important human pathogen, the first sRNAs to be described were found in the regulon of the CiaRH two-component regulatory system. Five of these sRNAs were detected and designated csRNAs for cia-dependent small RNAs. CiaRH pleiotropically affects β-lactam resistance, autolysis, virulence, and competence development by yet to be defined molecular mechanisms. Since CiaRH is highly conserved among streptococci, it is of interest to determine if csRNAs are also included in the CiaRH regulon in this group of organisms consisting of commensal as well as pathogenic species. Knowledge on the participation of csRNAs in CiaRH-dependent regulatory events will be the key to define the physiological role of this important control system.

**Results:**

Genes for csRNAs were predicted in streptococcal genomes and data base entries other than *S. pneumoniae *by searching for CiaR-activated promoters located in intergenic regions that are followed by a transcriptional terminator. 61 different candidate genes were obtained specifying csRNAs ranging in size from 51 to 202 nt. Comparing these genes among each other revealed 40 different csRNA types. All streptococcal genomes harbored csRNA genes, their numbers varying between two and six. To validate these predictions, *S. mitis*, *S. oralis*, and *S. sanguinis *were subjected to csRNA-specific northern blot analysis. In addition, a csRNA gene from *S. thermophilus *plasmid pST0 introduced into *S. pneumoniae *was also tested. Each of the csRNAs was detected on these blots and showed the anticipated sizes. Thus, the method applied here is able to predict csRNAs with high precision.

**Conclusions:**

The results of this study strongly suggest that genes for small non-coding RNAs, csRNAs, are part of the regulon of the two-component regulatory system CiaRH in all streptococci.

## Background

Post-transcriptional regulation by small RNAs (sRNAs) in bacteria is now recognized as an important regulatory mechanism modulating a wide range of physiological responses [1]. While a few bacterial sRNAs were known for quite some time, their prevalence and importance were not initially appreciated. In recent years impressive technical advances have led to the prediction and/or characterization of many novel sRNAs in various bacteria [2-4]. In *Escherichia coli *alone close to hundred sRNAs have been verified and new studies using deep sequencing of cDNAs (RNA-Seq) in *Vibrio cholerae *suggested the number of sRNAs to be several hundred [5]. Even in low-GC Gram-positive bacteria, where relatively few sRNAs had been detected [6,7], the numbers are steadily increasing [8-10].

In streptococci, information regarding sRNAs is so far restricted to two streptococcal species. Three sRNAs, fasX [11], *pel *[12], and *rivX *[13], have been detected serendipitously in *Streptococcus pyogenes*. A genome-wide study using tiling microarray and northern analysis added fourteen sRNAs in this organism [14].

In *Streptococcus pneumoniae*, the first sRNAs to be described in this bacterium were found in the regulon of the two-component regulatory system (TCS) CiaRH [15]. These sRNAs, designated csRNAs (**c**ia-dependent **s**mall RNA), are transcribed from the five strongest promoters of the CiaRH regulon. They show a high degree of similarity to each other, especially in the unpaired region between the two stem-loop structures characteristic for these sRNAs. Complementarity to the Shine-Dalgarno (SD) [16] sequence and the start codon AUG within this unpaired region suggested that the csRNA could control translation initiation of mRNAs. More recently, nine additional sRNA have been detected in *S. pneumoniae *D39, but their regulation has not been studied in greater detail [17]. A tiling array approach using RNA from *S. pneumoniae *TIGR4 suggested the existence of 36 sRNAs, but none was validated by northern blot analysis [18]. While the D39 work identified only csRNA1, all five csRNAs were detected in TIGR4. Both studies produced relatively few common sRNAs indicating a need for more efficient prediction methods.

The TCS CiaRH had been identified in a screen aimed at isolating spontaneous mutants of *S. pneumoniae *resistant to cefotaxime [19]. Besides β-lactam resistance, CiaRH affects lytic processes, genetic competence, bacteriocin production, and virulence [20-25]. Transcriptional mapping, *in vitro *CiaR binding, and *in vivo *expression studies, identified a direct repeat sequence, TTTAAG-N5-TTTAAG, to be essential for CiaR-mediated gene regulation [15]. The response regulator CiaR in *S*. *pneumoniae *controls 15 promoters leading to the expression of 24 genes, which are organized in 5 operons and 10 monocistronic transcriptional units.

CiaRH is also found in other streptococcal species and a number of studies have been performed aimed at elucidating the role of CiaRH in these organisms. In *Streptococcus mutans*, the system is involved in bacteriocin production, competence regulation, biofilm formation, and tolerance to environmental stresses [26-30]. Stress tolerance is also affected in CiaRH mutants of *Streptococcus gordonii *[31]. In *Streptococcus agalactiae*, intracellular survival and resistance to innate immunity defenses are diminished in CiaR-deficient strains [32]. Transcriptome studies using microarrays in *S. agalactiae *and *S. pyogenes *revealed genes regulated by the CiaRH TCS, but no overlap with the well defined CiaR regulon of *S. pneumoniae *was detected [32,33]. In *S. mutans *however, expression of *htrA*, encoding a serine protease and one of the genes most strongly regulated by CiaR in *S. pneumoniae*, is upregulated in a *ciaH *mutant strain [26]. In most of these streptococci the binding site for CiaR has not been identified and the genes directly controlled by CiaR are still not known. Most recently, the CiaR binding site in *S. mutans *has been defined to be NTTAAG-N5-WTTAAG [34], which is in perfect agreement with the one determined for *S*. *pneumoniae*.

In the present communication, we set out to determine whether genes for small non-coding RNAs are always part of the CiaR regulon in streptococci. Predictions of such sRNA genes were made for those streptococcal species whose complete genome sequences were available. To validate these predictions, expression of csRNAs in *Streptococcus mitis*, *Streptococcus oralis*, *Streptococcus sanguinis*, and from a *Streptococcus thermophilus *plasmid was tested by northern blot analysis. The results of these experiments strongly suggest that genes for sRNAs are contained in all CiaR regulons of streptococci.

## Results

### Conservation of the response regulator CiaR in streptococcal species

The two-component regulatory system CiaRH is present in all streptococcal species but is apparently not found in other bacteria. Searches using the histidine kinase CiaH of *S. pneumoniae *or its extracytoplasmic sensor domain identified homologues in streptococci but not in other groups of Gram-positive bacteria such as *Bacillus*, *Listeria*, *Staphylococcus*, *Lactobacillus*, or *Lactococcus *[35]. The streptococcal CiaH kinase proteins share between 47 and 86% identical residues (Table 1). The cognate response regulators, however, are much more similar showing between 80 and 99% identities (Table 1). CiaR belongs to the OmpR family of winged-helix transcription factors interacting with DNA by the recognition helix and the wings [36,37]. The recognition helix contacts particular DNA bases in the major groove and is thus determining the sequence specificity. The recognition helix of CiaR, positioned from aa 189 to 201 in the *S. pneumoniae *protein, is extremely conserved among all streptococcal CiaR response regulators (Table 1). Only valine to isoleucine replacements are detected at two positions suggesting that all CiaR regulators could bind to very similar DNA sequences. From these considerations we concluded that CiaR response regulators from streptococci should also bind to the sequence TTTAAG-N5- TTTAAG as determined for CiaR from *S. pneumoniae *[15].

**Table 1:** Similarity of CiaRH proteins of various streptococci to S. pneunomiae CiaRH proteins.

*Streptococcus*	**CiaR**^**a**^	**CiaH**^**b**^	**CiaR recognition helix**^**c**^
			VVEVYVSKVRKKL^d^
*S. mitis *B6	99	86	VVEVYVSKVRKKL
*S. oralis *Uo5	96	83	VVEVYVSKVRKKL
*S. sanguinis *SK36	88	60	VVEVYVSKIRKKL
*S. agalactiae *NEM316	88	51	VVEVYVSKVRKKL
*S. dysgalactiae *subsp. *equisimilus *GGS_124	84	50	VVEVYVSKIRKKL
*S. equi *subsp.*equi *4047	80	50	VVEVYVSKIRKKL
*S. equi *subsp. *zooepidemicus *MGCS10565	80	51	VVEVYVSKIRKKL
*S. gallolyticus *UCN34	81	51	VVEVYVSKIRKKL
*S. gordonii *str. Challis substr. CH1	89	65	VVEVYVSKIRKKL
*S. mutans *UA159	89	55	VVEVYVSKIRKKL
*S. pyogenes *MGAS315	84	51	VVEVYISKIRKKL
*S. suis *05ZYH33	88	48	VVEVYVSKIRKKL
*S. thermophilus *CNRZ1066	86^e^	47^e^	VVEVYVSKIRKKL
*S. uberis *0140J	84	51	VVEVYISKIRKKL
	OmpR	*E. coli*	SIDVQISRLRRMV

^a,b ^Similarity is given in% identical residues of *S. pneumoniae *R6 CiaR and CiaH, respectively.

^c ^The CiaR recognition helix was identified according to the OmpR family comparison defining DNA binding sites [37].

^d ^The recognition helix of *S. pneumoniae *R6 CiaR is shown for comparison. Residues deviating from CiaR R6 are underlined.

^e ^*ciaR *is interupted by a stop codon and was artificially fused for comparison. *ciaH *is interrupted by two frame shifts and was artificially fused for comparison.

The *ciaRH *genes in *S*. *thermophilus *are apparently not functional. In all *S*. *thermophilus *genome sequences available at the moment, *ciaR *is interrupted by a stop codon, while translation of full length *ciaH *is prevented by two frame shifts. A functional CiaRH system may not be necessary in *S*. *thermophilus *due to its adaptation to the dairy niche, which appears to be mainly achieved by loss-of-function mutations [38].

### Prediction of csRNAs in streptococci other than *S. pneumoniae*

To determine whether genes for csRNAs are present in other streptococcal species BLAST searches [39] of the nucleotide collection (nr/nt) data base were performed using the five pneumococcal csRNAs as query. The complete set of csRNA genes was detected in all *S*. *pneumoniae *genomes. Several hits were also obtained in whole genome sequences of streptococcal species other than *S. pneumoniae*, some of which representing most likely full-length csRNA genes, but most of the hits covered far less than 50% of the query. In addition, limited similarity to csRNAs was also detected in mobile DNA elements such as streptococcal phages or an *S. thermophilus *plasmid.

From these analyses it appeared likely that at least some csRNAs genes are present in streptococcal genomes, but the significance of short stretches of csRNA similarity was not clear. They could indicate more diverse csRNAs, much shorter versions or remnants of csRNAs, or they could be obtained simply by chance. To distinguish these possibilities, the presence of CiaR-controlled promoters should be indicative. These promoters should be located in intergenic regions and be followed by transcriptional terminators. Therefore, such arrangements were searched in selected streptococcal genome sequences and sequence entries showing limited similarity to csRNA genes mentioned above. For whole genomes, one strain of each species available from the NCBI microbial genome database was chosen for analysis and two genome sequences of oral streptococci recently completed in our laboratory were also included. In total, 14 streptococcal genomes were searched revealing the presence of 58 candidates for csRNA genes (Table 2), four csRNA genes on average per streptococcal species. While for three species, *S. sanguinis, S. gallolyticus*, and *S. gordonii*, six csRNA genes were predicted, *S. equi *subsp. *equi *apparently harbors only two. Three additional csRNA genes were predicted in other GenBank entries, two in *S. mitis *and one on plasmid pST0 from *S. thermophilus*. [40,41] These analyses also revealed that a number of shorter hits were not significant.

**Table 2:** Genes for csRNAs predicted in streptococcal species other than S. pneumoniae.

**strain**^**a**^	**csRNA**^**b**^	**size**^**c**^	begin	end
*S. mitis *B6	csRNA3(B6)	99	23857	23955
	csRNA4(B6)	96	1932077	1931982
	csRNA2(B6)	97	1936359	1936263
	csRNA1(B6)	94	1936548	1936455
	csRNA5(B6)	146	1944227	1944082
				
*S. oralis *Uo5	csRNA3(Uo5)	100	21777	21876
	csRNA4(Uo5)	93	1712653	1712561
	csRNA2(Uo5)	98	1714371	1714274
	csRNA6(Uo5)	200	1714673	1714474
	csRNA1(Uo5)	95	1724567	1724473
				
*S. sanguinis *SK36	csRNA1-1(SK36)	91	65114	65204
	csRNA1-2(SK36)	94	65322	65415
	csRNA7(SK36)	85	67110	67194
	csRNA8(SK36)	177	545784	545959
	csRNA2(SK36)	95	2160890	2160796
	csRNA1-3(SK36)	84	2183995	2183912
				
*S. agalactiae *NEM316	csRNA10(NEM316)	145	459709	459565
	csRNA11(NEM316)	96	622777	622872
	csRNA12(NEM316)	65	1755431	1755367
	csRNA13(NEM316)	66	2160062	2160127
				
*S. dysgalactiae *subsp. *equisimilus *GGS_124	csRNA14(GGS124)	68	1514790	1514723
	csRNA15(GGS124)	141	1916876	1917016
	csRNA16(GGS124)	127	1975022	1974896
	csRNA17(GGS124)	117	1975620	1975736
				
*S. equi *subsp. *equi *4047	csRNA18(4047)	50^d^	194835	194786
	csRNA17(4047)	105	200539	200643
				
*S. equi *subsp. *zooepidemicus *MGCS10565	csRNA18(MGCS10565)	67	159514	159448
	csRNA19(MGCS10565)	105	164153	164257
	csRNA20(MGCS10565)	108	236846	236739
				
*S. gallolyticus *UCN34	csRNA18(UCN34)	66	2864	2929
	csRNA40-1(UCN34)	65	51782	51718
	csRNA9(UCN34)	63	679884	679822
	csRNA38(UCN34)	138	2175160	2175297
	csRNA39(UCN34)	118	2189828	2189945
	csRNA40-2(UCN34)	71	2266270	2266200
				
*S. gordonii *str. Challis substr. CH1	csRNA7(CH1)	88	116691	116778
	csRNA21(CH1)	58	161804	161861
	csRNA2-1(CH1)	95	179826	179920
	csRNA1(CH1)	87	1360105	1360019
	csRNA22(CH1)	202	1721745	1721593
	csRNA2-2(CH1)	95	2038879	2038785
				
*S. mutans *UA159	csRNA23-1(UA159)	79	44425	44503
	csRNA24(UA159)	152	303857	303706
	csRNA23-2(UA159)	81	1501299	1501219
				
*S. pyogenes *MGAS315	csRNA15(MGAS315)	142	188268	188127
	csRNA14(MGAS315)	68	1276812	1276745
	csRNA25(MGAS315)	129	1784361	1784233
				
*S. suis *05ZYH33	csRNA26(05ZYH33)	172	52590	52761
	csRNA27(05ZYH33)	73	278732	278804
	csRNA28(05ZYH33)	51	352037	352094
				
*S. uberis *0140J	csRNA29(0140J)	84	46527	46610
	csRNA30(0140J)	83	54985	55067
	csRNA31(0140J)	67	64276	64210
	csRNA32(0140J)	140	1675433	1675572
				
*S. thermophilus *CNRZ1066	csRNA33(CNRZ1066)	66	8588	8653
	csRNA34(CNRZ1066)	85	42534	42618
	csRNA35(CNRZ1066)	64	44784	44847
	csRNA36(CNRZ1066)	97	51297	51393
	csRNA37(CNRZ1066)	127	51629	51755
				
*S. thermophilus *St0 plasmid pSt0	csRNA9(pST0)	60	677	736
				
*S. mitis *SF100	csRNA2(SF100)	98	2967	2870
	csRNA6(SF100)	200	3271	3072

^a ^The sequences used for the searches have the following accession numbers: *S. mitis *B6 [GenBank:FN568063]; *S. oralis *Uo5 [GenBank:FR720602]; *S. sanguinis *SK36 [GenBank:CP000387];*S. agalactiae *NEM316 [GenBank:AL732656]; *S*. d*ysgalactiae *subsp.*equisimilus *GGS_124 [GenBank:AP010935]; *S. equi *subsp. *equi *4047 [GenBank:FM204883]; *S. equi *subsp. *zooepidemicus *MGSC10565 [GenBank:CP001129]; *S. gallolyticus *UCN34 [GenBank:FN597254]; *S. gordonii *str. Challis substr. CH1 [GenBank:CP000725]; *S. mutans *UA159 [GenBank:AE014133]; *S. pyogenes *MGAS315 [GenBank:AE014074]; *S. suis *05ZYH33 [GenBank:CP000407]; *S. uberis *0140J [GenBank:AM946015]; *S. thermophilus *CNRZ1066 [GenBank:CP000024]; *S. thermophilus *St0 plasmid pSt0 [GenBank:AJ242480]; *S. mitis *SF100 [GenBank:AY007504].

^b ^csRNAs are considered the same type if at least 80% of the residues are identical with consecutive insertions/deletions of less than three nucleotides. If the same type is present in one strain, the csRNAs are designated -1 and -2, respectively.

^c ^Size is given in nucleotides assuming the transcriptional start seven bases downstream of the -10 region and the last U of the transcriptional terminator as the stop site.

^d ^csRNA gene is truncated by an insertion sequence.

For *S. agalactiae*, *S. equi *subsp. *zooepidemicus*, *S. mutans*, *S. pyogenes*, and *S. thermophilus *the genome sequences of more than one strain are available. To determine if the newly identified csRNA genes listed in Table 2 are also present in these strains, Blast searches were performed. All csRNA genes defined in one strain of a streptococcal species are detected in the other strains with one exception. The gene for csRNA14(MGAS315) of *S. pyogenes *MGAS315 was only found in four out of thirteen *S. pyogenes *strains, but an identical copy is contained in *S. dysgalactiae *subsp. *equisimilus *GGS_124 (Table 2).

**Figure 1:**
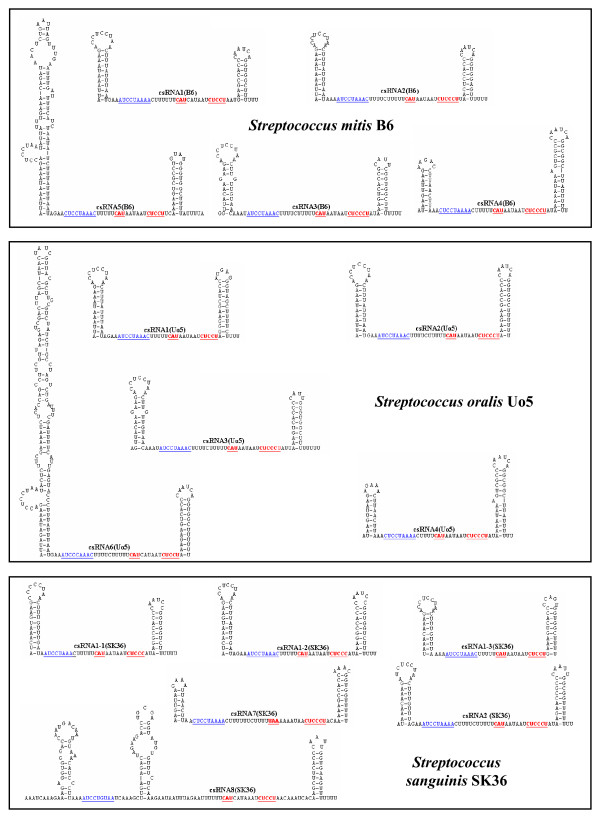
**Secondary structures of csRNAs from *S. mitis *B6, *S*. *oralis *Uo5, and *S. sanguinis *SK36**. The csRNA gene predictions from Table 2 were folded using the Mfold program [54]. Sequences complementary to Shine-Dalgarno [16] sequences or translation initiation codons are underlined and shown in red. A conserved stretch of nucleotides found in virtually all csRNAs is underlined and shown in blue. These csRNAs were verified by northern blot analysis (Figure 6).

**Figure 2:**
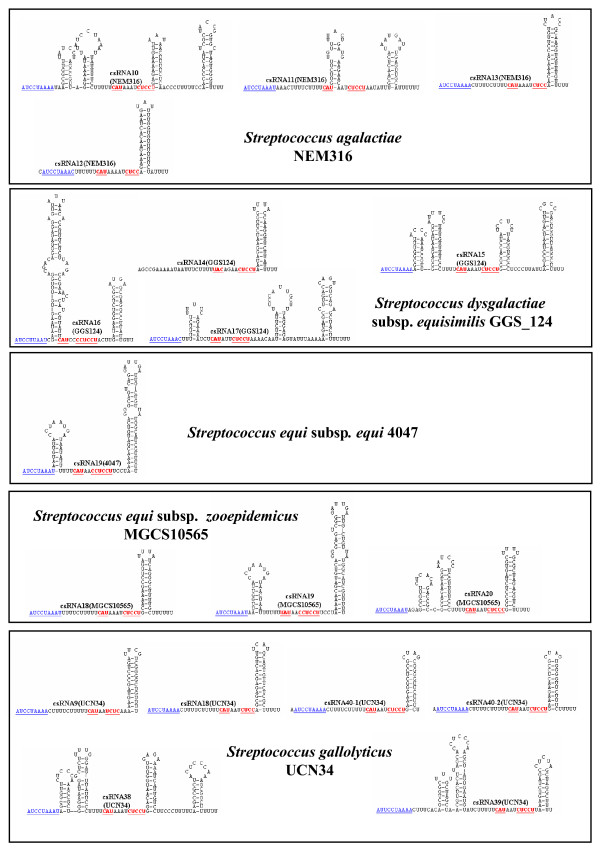
**Secondary structures of csRNAs from *S*. *agalactiae *NEM316, *S. dysgalactiae *subsp. *equisimilus *GGS_124, *S. equi *subsp. *equi *4047, and *S. equi *subsp. *zooepidemicus *MGCS10565, and *S. gallolyticus *UCN34**. The csRNA gene predictions from Table 2 were folded using the Mfold program [54]. Sequences complementary to Shine-Dalgarno [16] sequences or translation initiation codons are underlined and shown in red. A conserved stretch of nucleotides found in virtually all csRNAs is underlined and shown in blue.

**Figure 3:**
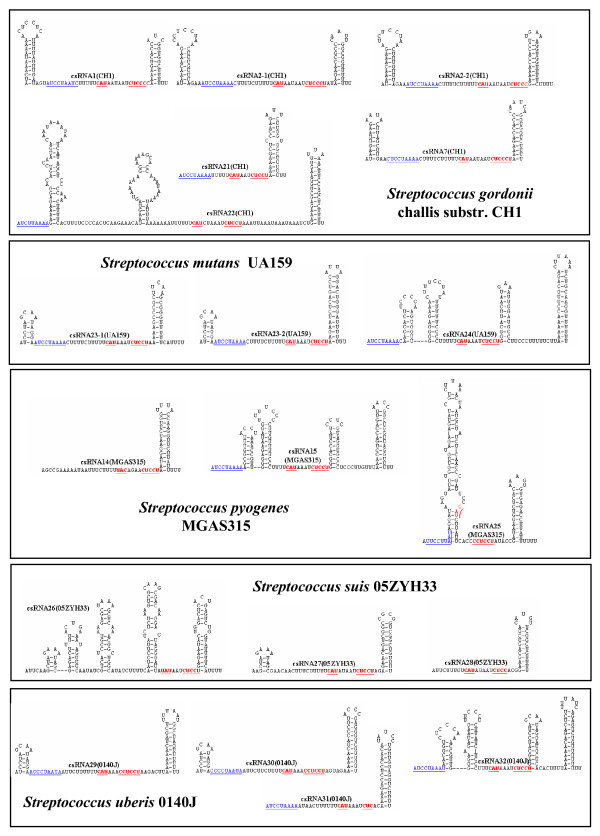
**Secondary structures of csRNAs from *S. gordonii *challis substr. CH1, *S. mutans *UA159, *S. pyogenes *MGAS315, *S. suis *05ZYH33, and *S. uberis *0140J**.. The csRNA gene predictions from Table 2 were folded using the Mfold program [54]. Sequences complementary to Shine-Dalgarno [16] sequences or translation initiation codons are underlined and shown in red. A conserved stretch of nucleotides found in virtually all csRNAs is underlined and shown in blue.

**Figure 4:**
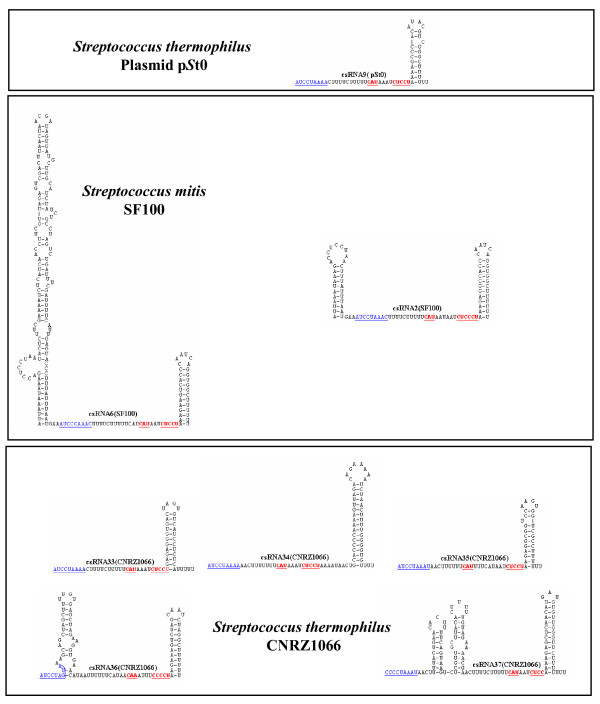
**Secondary structures of csRNAs from *S. thermophilus *plasmid pSt0, *S. thermophilus *CNRZ1066, and *S. mitis *SF100**. The csRNA gene predictions from Table 2 were folded using the Mfold program [54]. Sequences complementary to Shine-Dalgarno [16] sequences or translation initiation codons are underlined and shown in red. A conserved stretch of nucleotides found in virtually all csRNAs is underlined and shown in blue. *S. thermophilus *csRNA are not expressed in their native hosts due to *ciaR *inactivation.

Comparisons of the csRNA genes among each other revealed a complex picture. Short stretches of conserved sequences are present in all genes, but overall a surprising diversity was observed. If a gene showed 80% identity to another one and had no insertions/deletions of more than three consecutive nucleotides, the two genes were considered to specify the same RNA. Applying these criteria, genes for 40 different csRNA types were predicted, which varied in size from 51 to 202 nt. Secondary structure predictions revealed 32 csRNAs exhibiting two stem-loop structures similar to the csRNAs known from *S. pneumoniae*, 16 with only the termination stem-loop, and 13 with more complex structures (Figures 1, 2, 3 and 4). In all csRNAs, sequences are present that could potentially base pair to translation initiation regions of mRNAs.

**Figure 5:**
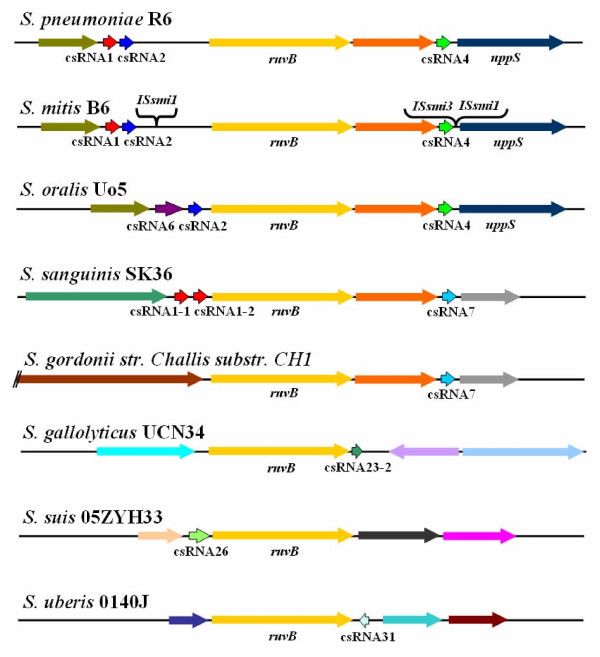
**Genomic locations of csRNAs in diverse streptococcal species**. The *ruvB*- *uppS *region of *S*. *pneumoniae *harboring genes for csRNA1, csRNA2, and csRNA4 is compared with the corresponding *ruvB*-containing loci from *S. mitis*, *S. oralis*, *S. sanguinis*, *S. gordonii*, *S*. *gallolyticus*, *S. suis*, and *S. uberis*. Genes with high identity are shown in the same color.

Corresponding to the great variety of csRNA sequences, the genomic locations of csRNA genes are also variable. As an example, the *ruvB*-*uppS *region of *S. pneumoniae *is shown in Figure 5 harboring csRNA genes for csRNA1, 2, and 4. In *S. mitis *B6, this region including csRNA genes is conserved but disrupted by insertion elements. In *S. oralis *Uo5, csRNA6 is found instead of csRNA1. *S. sanguinis *SK36 still harbors three csRNA genes, but csRNA7 replaces csRNA4. The other species, *S. gordonii *CH1, *S. gallolyticus *UCN34, *S. suis *05ZYH33, and *S. uberis *0140J have only one csRNA gene at variable positions and orientations. The other streptococcal species listed in Table 1 do not harbor csRNA genes at this locus. The amazingly variable genetic context of csRNA genes is consistent with the proposed role of the csRNAs as trans-acting post-transcriptional effectors.

### Detection of csRNAs in *S. mitis*, *S. oralis*, and *S. sanguinis*

**Figure 6:**
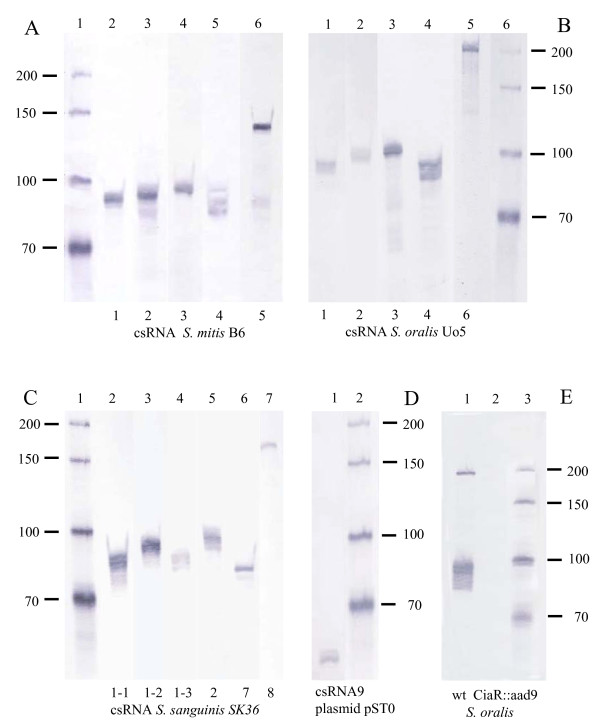
**Northern blot analysis to detect csRNAs in total RNA from streptococci**. **(A) Detection of csRNAs in *S. mitis *B6**. Northern blot analysis of total RNA isolated from *S. mitis *B6. RNAs were detected by hybridization of digoxigenin-labeled probes complementary to individual csRNAs as indicated at the bottom of the blot. **(B) Detection of csRNAs in *S. oralis *Uo5**. Northern blot analysis of total RNA isolated from *S. mitis *B6. RNAs were detected by hybridization of digoxigenin-labeled probes complementary to individual csRNAs as indicated at the bottom of the blot. **(C) Detection of csRNAs in *S*. *sanguinis *SK36**. Northern blot analysis of total RNA isolated from *S. mitis *B6. RNAs were detected by hybridization of digoxigenin-labeled probes complementary to individual csRNAs as indicated at the bottom of the blot. **(D) Detection of csRNA9 from plasmid pST0 cloned in *S. pneumoniae *lacking endogenous csRNAs**. Northern blot analysis of total RNA isolated from *S. mitis *B6. RNAs were detected by hybridization of digoxigenin-labeled probes complementary to individual csRNAs as indicated at the bottom of the blot. **(E) Dependence of csRNA expression on CiaR in *S. oralis *Uo5**. Northern blot analysis of total RNA isolated from *S. oralis *Uo5 wild type and a *ciaR::aad9 *mutant derivative. The blot was hybridized with digoxigenin-labeled probes complementary to all csRNAs. Lane 1: *S. oralis *Uo5 RNA. Lane 2: *S. oralis ciaR*::*aad9 *RNA. Lane 3: *In vitro *synthesized RNA size standard.

To validate the csRNA gene predictions by northern blot analysis, three streptococcal species, *S. mitis *B6, *S. oralis *Uo5, and *S. sanguinis *SK36 were chosen. The strains were grown in C-medium to the end of exponential growth phase, conditions applied to detect csRNAs in *S. pneumoniae *[15]. Total RNA was extracted and separated on denaturing polyacrylamide gels. Digoxigenin labeled probes were designed to detect single csRNA types in each strain. As shown in Figure 6, all predicted csRNAs could be verified in these northern analyses. While *S. mitis *B6 (Figure 6A) and *S. oralis *Uo5 (Figure 6B) expressed five csRNAs, *S. sanguinis *produced even six of them (Figure 6C). Three new csRNA types were detected, one in *S. oralis *(csRNA6) and two in *S. sanguinis *(csRNA7; csRNA8). The csRNAs showed the anticipated sizes indicating that the assumed starts and ends were correct. In virtually all cases more than one band was detected with one csRNA probe, which is due to termination of transcription at several positions within the poly(U) stretches, as demonstrated also for the csRNAs of *S. pneumoniae *[15]. The results of these northern analyses expand the experimentally proven csRNA types to eight.

Since *S. oralis *is readily transformable, a *ciaR *mutant strain was created by integrating a resistance marker into the gene. Subsequently, northern blot analysis was performed to examine csRNA expression in the ciaR mutant strain. Using probes for all *S. oralis *csRNAs, no signal was detectable in the CiaR-deficient mutant (Figure 6E). This result clearly demonstrates the dependence of csRNA expression on a functional CiaR response regulator.

### Expression of a csRNA gene from plasmid pST0

*S. thermophilus *appears to be unique among streptococci, since its CiaRH system is inactivated by mutations. The *ciaRH *genes in the three sequenced *S. thermophilus *strains CNRZ1066, LMG18311, and LMD9 harbor three identical mutations. Interestingly, a plasmid of another *S. thermophilus *strain ST0 contained a csRNA gene on a plasmid designated pST0 [41] Since this gene was the only one of the predicted csRNA genes that is not located in the genome and the small RNA represented a new type with only one stem-loop structure (csRNA9; Figure 4), we wanted to test its expression. To determine the status of the CiaRH system in that strain, the *ciaRH *region of *S. thermophilus *ST0 was amplified and sequenced. The same three mutations known from *S. thermophilus *whole genome sequences inactivating both *cia *genes were detected. Accordingly, using a csRNA9-specific probe did not reveal a signal on northern blots using RNA purified from *S. thermophilus *ST0 (data not shown). To provide a functional CiaRH system for csRNA9 expression, the gene, designated *ccnI*, was amplified from plasmid pST0 and cloned into the *S. pneumoniae *integration vector pMRT2-2 [42] as described in the Methods section. The resulting plasmid pMRT-*ccnI *was transferred to the *S. pneumoniae *strain RK12345 expressing no csRNAs [15]. Integration of *ccnI *occurred in the *bgaA *locus by double cross-over. RNA purified from that strain (RK12345; *bgaA::ccnI) *was subjected to northern blot analysis using a csRNA9-specific probe. As shown in Figure 6D, a band smaller than 70 nt was detected corresponding to the csRNA9 of 60 bp. Thus, *ccnI *is indeed expressed when a functional CiaRH system is provided. Consequently, introduction of *ccnI *into a CiaR-deficient strain (*ciaR*::*aad9*) did not result in csRNA9 production (data not shown). It is curious that although the CiaRH system is inactive in *S. thermophilus*, targets such as csRNA genes have still all necessary expression signals.

## Discussion

By searching data base entries with the consensus sequence for CiaR-activated promoters, 61 genes for csRNAs were predicted in 14 streptococcal species. 17 of these predictions were verified by visualizing csRNAs from *S. mitis *B6, *S oralis *Uo5, *S. sanguinis *SK36, and *S. thermophilus *plasmid pST0 on northern blots. In addition, a recent genome-wide analysis of sRNAs in *S. pyogenes *[14] identified two sRNAs in strain MGAS2221, which are in fact controlled by CiaR. These sRNAs, SR195750 and SR1719800, correspond to our predicted csRNA15(MGAS315) and csRNA25(MAGAS315), respectively. Their experimentally determined transcriptional start points match exactly the predicted ones for csRNA15 and csRNA25. Since the gene for csRNA14(MGAS315) is missing in MGAS2221, csRNA14 could not be detected in the study by Perez et al. [14]. The small RNA search in *S. pyogenes *and our work identified almost one third of the predicted csRNAs strongly suggesting that small RNAs controlled by the response regulator CiaR exist in all streptococci.

Both csRNAs in *S. pyogenes *are expressed in standard medium during exponential growth and are also detected in stationary phase. The same expression pattern was observed in *S. pneumoniae *[15] as well as in *S. mitis*, *S. oralis *and *S. sanguinis*, the streptococcal species used in our study (data not shown). It will be interesting to see if csRNAs will be expressed under similar conditions in all streptococci.

**Figure 7:**
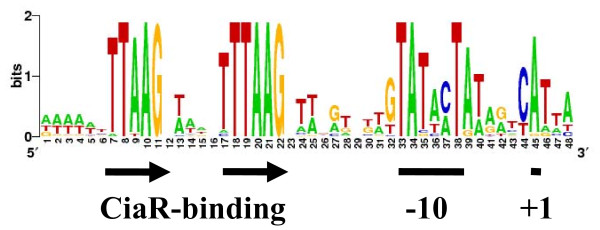
**Sequence logo of streptococcal promoters for csRNA genes shown to be expressed by northern blots**. The logo was produced by WebLogo [43] using a multiple alignment of promoter sequences of csRNA genes from *S. pneumoniae *R6, *S. mitis *B6, *S. oralis *Uo5, *S. pyogenes *MGAS2221, and plasmid pST0.

Together with the five csRNAs originally detected in *S. pneumoniae*, 24 csRNAs have been verified so far by northern blot analysis. Their promoters were therefore used to derive a new consensus sequence for strongly CiaR-activated promoters on a broader basis. To do so, a sequence logo was created by analyzing a multiple promoter sequence alignment by Weblogo [43]. As shown in Figure 7, the repeat sequence TTTAAG is clearly visible, especially in the second part of the repeat. Only the first T is variable, replaced only twice by A and C, respectively. The first T in the first repeat is far less conserved being present in only 11 promoters. In addition, the second T and the first A are slightly variable. In both repeats, the third T, the second A and the final G are invariable. Thus, the CiaR repeat consensus appears to be NTTAAG-N5-TTTAAG rather than TTTAAG-N5-TTTAAG as proposed earlier [15]. Besides the conservation of the -10 region there is a C preferentially found immediately upstream of the transcription initiation site. The significance of this observation is not clear at the moment.

The majority of sRNAs characterized to date exert their regulatory function by base pairing to mRNAs [1]. These sRNAs regulate translation and/or stability of target mRNAs, in most cases negatively. Quite often, access to the ribosome binding site is blocked by sRNA-mRNA base pairing. The csRNAs of *S. pneumoniae *and those characterized or predicted in this work show complementarity to translation initiation regions (TIR). They can potentially base pair with the Shine-Dalgarno (SD) sequence [16] including the following start codon (Figures 1, 2, 3 and 4). These anti-SD and anti-start codon sequences are located in unpaired regions of the csRNAs (Figures 1, 2, 3 and 4). In 53 of the 61 csRNAs, CAU is found potentially serving as an anti-AUG. In the remaining nine csRNAs, anti-GUG and anti-UUG sequences are present. The anti-TIR sequences in all csRNAs strongly suggest that these sRNAs bind to mRNAs to hinder access of ribosomes thereby blocking translation initiation. It will be interesting to determine and compare their molecular targets in diverse streptococcal species.

Although the potentiality to target TIRs of mRNAs is a common feature of the csRNAs, primary sequence conservation within the anti-TIR regions is rather limited. In addition, overall similarity of the csRNAs is poor. However, a short stretch of conserved nucleotides is found in almost all csRNAs (Figures 1, 2, 3 and 4). Sequences very similar to [A/C]UCCUAAA[A/C] originally defined for the csRNAs of closely related *S. pneumoniae *[15], *S. mitis*, and *S. oralis *are present in all but four csRNAs (Figures 1, 2, 3 and 4). Comparing all sequences indicated that the last position in this nonamer is less conserved than anticipated from the *S. pneumoniae*, *S. mitis*, and *S. oralis *comparison. The nonamer is located immediately downstream of the first stem-loop or at the 5'-end of the csRNAs (Figures 1, 2, 3 and 4). It is missing in csRNA14 and, curiously, in all csRNAs predicted in *S. suis*.

If blocking ribosome binding sites by anti-TIR sequences would be the primary mode of action of the csRNAs, the conserved sequence introduced above should have another function. A protein binding site would be an attractive hypothesis. The global post-transcriptional regulator Hfq is a key factor in sRNA-mRNA interaction and regulation especially in *E*. *coli *but also in quite a number of other bacteria [44,45]. In AT-rich Gram-positive bacteria however, a role of Hfq in sRNA-mediated regulatory events is less obvious [46,47] and was only recently demonstrated for the first time with one sRNA in *Listeria monocytogenes *[48]. In addition, the *hfq *gene is apparently lacking in enterococci, lactococci, lactobacilli, and streptococci [45]. Assuming that other protein(s) could substitute for Hfq in streptococci, the short conserved sequence in csRNAs may be part of a binding site for this factor.

## Conclusions

Here we have demonstrated that small RNAs predicted on the basis of a CiaR binding site and an appropriately spaced -10 region, which is followed by a transcriptional terminator, are expressed in *S. mitis*, *S. oralis*, *S. sanguinis *and from plasmid pST0. These data, together with published proof of expression of these sRNAs in *S. pneumoniae *and *S. pyogenes*, strongly suggest that genes for sRNAs belong to the regulon of the response regulator CiaR in all streptococcal species. Expression of the CiaR-dependent csRNAs and the associated post-transcriptional regulatory events may be the reason for the pleiotropic phenotypes caused by CiaR-dependent control. Elucidation of the molecular mechanisms of csRNA regulation appears to be crucial to understand the role of CiaR in streptococcal physiology. Hence, the suggestion of S. Gottesman to 'watch for small RNAs in all your favourite regulatory circuits' [49] is especially rewarding for the CiaR regulon in streptococci.

## Methods

### Prediction of csRNA genes

To detect csRNA genes in organisms other than *S. pneumoniae*, BLAST [39] searches were performed using the nucleotide collection (nt/nr) database at NCBI. Only very few full-length hits were obtained, but quite a number of short stretches of similarity was detected. Thus, simply looking for similarity did not appear to be adequate to identify csRNA genes.

We therefore applied a more specific search using the known structure of CiaR-activated promoters in combination with transcriptional terminators. The promoters were predicted with Motif search and terminators were identified with TransTermHP. Both programs are combined in a service designated the Non-coding RNA Gene Finder available at [50]. Motif search is also separately available at [51]. It uses Rnabob to find motifs in genomic DNA. Rnabob is a program freely available from [52]. The transcriptional terminator prediction program has been described [53]

The RNA gene finder first finds user defined patterns and subsequently searches for transcriptional terminators in a user defined distance. The candidate RNA is then folded by means of Mfold [54].

The csRNAs listed in Table 2 were predicted using TTTAAG for the first two parts of motif search allowing one mismatch each. The distance between the two TTTAAG stretches was set to five. The third part represented the -10 region TATAAT. Two mismatches were allowed and the distance to the last TTTAAG was ten. The terminator structure was searched in a distance of 250 to the -10 region. Results obtained in this way were inspected and hits showing a mismatch at the G positions within TTTAAG were discarded, since it has been shown, that this residue is extremely important for efficient CiaR-mediated gene activation [15]. The predicted csRNAs were folded using Mfold [54] allowing no wide-range interactions that are more than 50 nucleotides apart. The results of these secondary structure predictions are shown in Figures 1, 2, 3 and 4. For nearly all csRNAs exceeding 100 bases in length, more than one structure was predicted. In these cases, the most stable structure is shown. The corresponding promoter sequences are listed in the additional file 1.

The DNAs that were searched included streptococcal whole genome sequences listed in Table 1 and those GenBank entries that yielded short BLAST hits with csRNA sequences from *S. pneumoniae*. With the csRNA genes detected in this way, BLAST searches were again performed, but yielded no further csRNA candidates. It appears, however, that incomplete remnants of csRNAs are present in some data base entries.

### Bacterial strains and growth conditions

The streptococcal species *S. mitis *B6 [55], *S. oralis *Uo5 [56] and *S. sanguinis *SK36 [57] were used for RNA isolation to detect csRNAs. From *S. thermophilus *ST0 [41], plasmid pST0 was purified. *S. pneumoniae *RK12345 [15], a derivative of S. pneumoniae R6 [58] devoid of all five csRNAs served for integrational cloning of *ccnI *from plasmid pST0. RCR1, a *ciaR *mutant of *S. pneumoniae *R6 was used to confirm the CiaR dependency of *ccnI *expression. *Escherichia coli *DH5α [Φ80 d*lacZ*ΔM15 Δ(*lacZYA-argF*) *recA1 endA1 hsdR17 supE44 thi-1 gyrA96 phoA relA1*] was used for cloning procedures.

Streptococci were grown at 37°C without aeration; *S. mitis*, *S. oralis*, and *S. pneumoniae *in C+Y medium [59]*S. sanguinis *in Brain Heart Infusion broth, and *S. thermophilus *in LM17 medium (0.5% Lactose). *E. coli *was grown aerobically in LB-medium.

### Integration of *ccnI *into the genome of *S. pneumoniae*

Since *S. thermophilus *is a natural *ciaRH *mutant, the gene *ccnI*, specifying csRNA9 and located on *S. thermophilus *pST0, should be expressed in *S. pneumoniae*. For that purpose, the integrative vector pMRT2-2 was applied, which is the ancestor of the reporter plasmids pTP1 and pPP2 without *E. coli lacZ *[42,60]. The region of the putative csRNA gene *ccnI *present on pST0 was amplified by a PCR Primer pair containing *BamH*I and *Sph*I sites, respectively (cgcggatccGCAGACAATAGCACTCGTATAGATG; ggcgcatgcCATTTATCCGTGCGTCATCG). PCR amplification and cloning in *E. coli *was carried out by standard procedures as described [15]. The resulting plasmid pMRT2-2-*ccnI *was introduced into *S. pneumoniae *RK12345 as described [15]. After transformation of RK12345 with pMRT2-2-*ccnI*, correct integration of *ccnI *into the *bgaA *locus in the *S. pneumoniae *genome was confirmed by PCR and DNA sequencing. The *ccnI*-containing plasmid was also used to transform RCR1, a *ciaR *mutant of *S. pneumoniae *R6. RNA from both strains with integrated *ccnI *was isolated and subjected to northern blot analysis.

### RNA purification and northern blot analysis

The strains were grown in the media mentioned above to an OD_600 _of 0.8 corresponding to late logarithmic growth phase. RNA purification and northern blot analysis was carried out as described previously [15] Synthetic oligonucleotides specific for individual csRNAs were labeled with digoxigenin-ddUTP and terminal transferase as described in the DIG Oligonucleotide 3'-end labeling kit instructions (Roche). Oligonucleotides used for csRNA detection are listed in the additional file 2. The following nucleotide, CCACTAGTTCTAGAGCCGGCCGCCACCGCGGTGGAGCTCCAATTCGCCC, was applied to visualize the RNA size standard prepared as described [15].

### Construction of a *ciaR *mutant strain of *S. oralis *Uo5

To inactivate the *ciaR *gene in *S. oralis *Uo5, the *ciaR::aad9 *region from *S. pneumoniae *RCR1 was amplified by primers located 1 kb up- and downstream of the *aad9 *insertion site. Due to the high similarity of the *ciaRH *region in *S. pneumoniae *and *S. oralis *Uo5 (around 90% identity) it was expected that the *S. pneumoniae *DNA should be integratable into *S. oralis*. Competent *S. oralis *cells were obtained according to the *S. pneumoniae *procedure and selection with 80 μg/ml spectinomycin readily yielded transformants with disrupted *ciaR*.

## Authors' contributions

PM and MK performed the experiments. RH provided strains and genome sequences. MN performed bioinformatic analyses. RB designed research and wrote the manuscript. All authors read and approved the final manuscript.

## Supplementary Material

Additional file 1 **Alignment of csRNA promoters from streptococci**. All predicted CiaR-dependent promoters driving expression of small non-coding csRNAs are shown. Click here for file

Additional file 2 **Oligonucleotides used to detect csRNAs**. Oligonucleotides used as probes to detect csRNAs are listed. Click here for file
