# Supplementary material for: Identification of genes for small non-coding RNAs that belong to the regulon of the two-component regulatory system CiaRH in Streptococcus
Source: BMC Genomics. 2010 Nov 24;11:661. doi: 10.1186/1471-2164-11-661 (PMC3091779; doi:10.1186/1471-2164-11-661)
Supplement: Additional file 1 — Alignment of csRNA promoters from streptococci. All predicted CiaR-dependent promoters driving expression of small non-coding csRNAs are shown. [file 1471-2164-11-661-S1.DOC]

**Streptococcal csRNA gene promoters.**

***S. mitis* B6**

PcsRNA3(B6) TTTTTAAGATTTGTTTAAGATAGGTCGAG**TATACT**ATAGACA

PcsRNA4(B6) TACTTAAGGAAACTTTAAGCTAGGGAGTG**TATACT**AAGTCCA

PcsRNA2(B6) AAGTTAAGCTAGTTTTAAGCTTCGTCATG**TATCAT**ATAGTTA

PcsRNA1(B6) TTCTTAAGCAAAGTTTAAGGATGATCTAG**TATTAT**ATAGTCA

PcsRNA5(B6) TTTTTAAGCTCTATTTAAGGTTGGATGTG**TATACT**ATAATCA

***S. oralis*Uo5**

PcsRNA3(Uo5) AATTTAAGTTAGCTTTAAGATTCATCTTG**TATTAT**ATAATCA

PcsRNA4(Uo5) AACTTAAGAAAAATTTAAGTTAGGAACGG**TATACT**AAGTCCA

PcsRNA2(Uo5) AAGTTAAGTTAGCTTTAAGATTCATCTTG**TATTAT**ATAATCA

PcsRNA6(Uo5) TTATTAAGTAAAGTTTAAGTCTCCTCGTG**TATTAT**ATAGTTA

PcsRNA1(Uo5) GATTTAAGTTGAGTTTAAGGATGACTGTG**TATACT**ATAATCA

***S. sanguinis* SK36**

PcsRNA1-1(SK36) ACTTTAAGTTTTTCTTAAGCTAAGAGCAG**TATACT**ATGAGTA

PcsRNA1-2(SK36) AAGTTAAGGTTTCTTTAAGTTAGGGGCAG**TATACT**ATGAGTA

PcsRNA7(SK36) ATTTTAAGGAAGTTTTAAGAAATGGATAT**TATACT**ATAACCA

PcsRNA8(SK36) TTTATAAGTAGGCTTTAAGTTTGAGGGAT**TATACT**ATTGTCA

PcsRNA2(SK36) ACTTTTAGGAAAATTTAAGATAAGTGTTC**TATACT**AAGACCA

PcsRNA1-3(SK36) ATCTTAAGATTCATTTAAGATATATATTT**TATACT**AACGGCA

**Plasmid pST0 *S. thermophilus***

PcsRNA9(pST0) AGATTAAGACTTTATTAAGCGGCCTTTTT**TATAAT**ATAGTCA

***S. agalactiae* NEM316**

PcsRNA10(NEM316) TTTTTAAGGTTTATTTAAGGTTTCTACTG**TATACT**AGTATCA

PcsRNA11(NEM316) TATTTAAGTTTTGTTTAAGGTGAACTTTG**TATACT**TTAAATA

PcsRNA12(NEM316) CTTTTAAGGAAACGTTAAGTAAGGGCGGT**TATACT**ATAAACA

PcsRNA13(NEM316) GTTTTAAGACTTGTTTAAGCATGGCCTTA**TATACT**ATAACCA

***S. dysgalactiae* subsp. equisimilus GGS_124**

PcsRNA14(GGS124) caattaagctaggtttaagttttctaagc**taaacc**acgctta

PcsRNA15(GGS124) gatttaaggaaagtttaaggttcctatcg**tagaat**aaaacca

PcsRNA16(GGS124) gaattaaggtttttttaagcctcttccct**tatact**aaggaca

PcsRNA17(GGS124) tgattaaggtttatttaaggtaggcactc**tatact**atccttA

***S. equi* subsp. *equi* 4047**

PcsRNA18(4047) cagttaagcttttcttaaggaaactagct**tatact**tacacta

PcsRNA17(4047) gaattaagctttctttaagcaagggtgtg**tatact**agccgta

***S. equi* subsp. *zooepidemicus* MGSC10565**

>PcsRNA18(MGSC10565)CAGTTAAGCTTTTCTTAAGGAAACTAGCT**TATACT**TACACTA

>PcsRNA19(MGSC10565)gagttaagctttctttaagcaagggtgtg**tatact**aaccata

>PcsRNA20(MGSC10565)AGGTTAAGAAAAGTTTAAGCTTTATGCTA**TACAAT**ATAACTA

***S. gallolyticus* UCN34**

PcsRNA18(UCN34) tctttaagaaaaatttaaggaagaattta**tacact**ataacca

PcsRNA23-1(UCN34) tttttaagaaaaaattaaggcacccgatt**tacaat**ataaaca

PcsRNA9(UCN34) cgtttaaggtttctttaaggtttctccga**tagaat**atagaca

PcsRNA38(UCN34) actttaaggtttctttaaggtttctccga**tatact**ataaaca

PcsRNA39(UCN34) tccttaagatttctttaaggctacctttt**tatact**ttagtca

PcsRNA23-2(UCN34) agcttaaggtaagtttaagcaagtcttta**tatact**ataaaca

***S. gordonii* str Challis substr. CH1**

PcsRNA7(CH1) GCTTTAAGATAATTTTAAGTTGGCTCGAG**TATACT**GTAGTCA

PcsRNA21(CH1) ACTTTAAGTTTTCTTTAAGTAAGGGAGTT**TATGAT**ATACCTA

PcsRNA2-1(CH1) GCTTTTAGGAAAGTTTAAGGTAAGAGATT**TATACT**AAGATCA

PcsRNA1(CH1) TCTTTAAGAATAATTTAAGATGCTTATTC**TATACT**ATAACCA

PcsRNA22(CH1) tctataagtcttccttaagctttgctggt**tatact**atattca

PcsRNA2-2(CH1) AGCTTAAGGATATTTTAAGTTAAAGCTCT**TATACT**GTAATCA

***S. mutans* UA159**

csRNA23-1(UA159) AGTTTAAGGAGTGATTAAGAAAGATCTTA**TATACT**ATAGTCA

csRNA24(UA159) ACTTTAAGGTTTCTTTAAGGTTTCTCATA**TATACT**TTAATCA

csRNA23-2(UA159) TTCTTAAGTTTCTTTTAAGAATCCTATCT**TATACT**ATAGTCA

***S. pyogenes* MGAS315**

PcsRNA15(MGAS315) CATTTAAGAAAACTTTAAGGTTACTATCG**TAGAAT**GAAAACA

PcsRNA14(MGAS315) caattaagctaggtttaagtttcctaagc**taaact**acgctta

PcsRNA25(MGAS315) ggcttaaggtttctttaagtcttatctag**tatact**aatgtca

***S. suis* 05ZYH33**

PcsRNA26(05ZYH33) TCTTTTAGTTTCCCTTAAGTTTGGCGAGT**TATACT**TTATTCA

PcsRNA27(05ZYH33) ATTTTCAGTTTCTTTTAAGTTTGTAGCTA**TATACT**ATATTCA

PcsRNA28(05ZYH33) AAGTTAAGGTTTGTTTAAGCATTGGAAGA**TACAAT**ATATTTA

***S. uberis* 0140J**

PcsRNA29(0140J) ACTTTAAGGTCTAATTAAGCAATGAATCT**TATACT**AGTACTA

PcsRNA30(0140J) tggttaaggtgcctttaagaaagctagtg**taagat**aaacata

PcsRNA31(0140J) ATTTTAAGCTATTTTTAAGTTTTTAAGGC**TATGAT**ATAATCA

PcsRNA32(0140J) GAGTTAAGAAATCTTTAAGGTTGATAGCT**TATGAT**AAGACTA

***S. thermophilus* CNRZ1066**

PcsRNA33(CNRZ1066) AACTTAAGGAAAGTTTAAGTAAATCTCTA**TATACT**ATAAAAC

PcsRNA34(CNRZ1066) GCCTTAAGCTTTCTTTAAGCTATCCAAGC**TATACT**AAGGTCA

PcsRNA35(CNRZ1066) AATTTAAGTCTCTCTTAAGCTATTTAGAC**TATACT**ATCATTA

PcsRNA36(CNRZ1066) ATTTTAAGTAACCTTTAAGTTCATTAAGA**TATATT**ATAACCA

PcsRNA37(CNRZ1066) GATTTAAGGTTTCTTTAAGGTAGGGAGGT**TATACT**GTAAATC

***S. mitis* SF100**

>PcsRNA2(SF100) aagttaagttagctttaagattcatcttg**tatcat**ataatca

>PcsRNA6(SF100) ttcttaagtaaagtttaagtctccttgtg**tattat**atagtta

The CiaR binding site TTTAAG-5-TTTAAG is shown in yellow. The -10 region is indicated in blue. Each sequence ends with the transcriptional start site. All csRNAs from *S. mitis* B6, *S. oralis*, *S. sanguinis* and plasmid pST0 have been shown to be expressed. In addition, csRNA15 and csRNA25 have been verified in *S. pyogenes*.
