# Supplementary material for: Identification of genes for small non-coding RNAs that belong to the regulon of the two-component regulatory system CiaRH in Streptococcus
Source: BMC Genomics. 2010 Nov 24;11:661. doi: 10.1186/1471-2164-11-661 (PMC3091779; doi:10.1186/1471-2164-11-661)
Supplement: Additional file 2 — Oligonucleotides used to detect csRNAs. Oligonucleotides used as probes to detect csRNAs are listed. [file 1471-2164-11-661-S2.PDF]

Oligonucleotides to detect csRNAs

csRNA	sequence
csRNA1(B6)	CCTGATTGGGTGACTTCATTAGGAGATTATG
csRNA2(B6)	CCTGATTGGGTGGAGTTAAGGGAGATTATTATG
csRNA3(B6)	TATTTGTTACAACAAGTTAGGAGGTCTTCTTGTAAC
csRNA4(B6)	GTTTTAGGAGTTTAAGTTAAGGTCTTCTTAACTTAT
csRNA5(B6)	GAAAATCAAATTCAAACTAAATCAGAGTTATCTGTTTCAAATAAT
csRNA1(Uo5)	CCAAAAGAAAAGCTACCCTCATGGTAGCTTCCCTAGG
csRNA2(Uo5)	CTGATTGGGTGGCTTCTTTA
csRNA3(Uo5)	GTTACAACAAGTTAGGAGGTCTTCTTGTAACT
csRNA4(Uo5)	AAAAGTTTTAGGAGTTTAAGTTAAGTTTCTCTTAACTTAT
csRNA5(Uo5)	GATAGACGCAAGCGTAACGATAGTTAGCTAAGTCGACTCT
csRNA1-1(SK36)	GGATTATCAAACAAAGTTAGGGGGCTT
csRNA1-2(SK36)	ATTTCTATCAAATAAAGTTAGGAGGTCT
csRNA1-3(SK36)	AAAGCCACCACTGGGGTGACTTTCAGGAGAT
csRNA2(SK36)	AAAAACCGCCAATTGGGCGGTTCTTATAGGG
csRNA7(SK36)	CCACCGTTTGGTGGCTTTTTGTAGGGAGA
csRNA8(SK36)	AATTCTAAATTATTCTTTGAGAAACACATACCTCGCA
csRNA9(p*St*0)	GACGTATGTGGGCTTTTAGGAGATTTATG

The oligonucleotides were labeled at the 3’-end with digoxigenin.
